# SUSD2 promotes tumor-associated macrophage recruitment by increasing levels of MCP-1 in breast cancer

**DOI:** 10.1371/journal.pone.0177089

**Published:** 2017-05-05

**Authors:** Elizabeth M. Hultgren, Mitch E. Patrick, Rick L. Evans, Catherine T. Stoos, Kristi A. Egland

**Affiliations:** 1 Cancer Biology Research Center, Sanford Research, Sanford School of Medicine of the University of South Dakota, Sioux Falls, South Dakota, United States of America; 2 Physicians Laboratory, Sioux Falls, South Dakota, United States of America; University of South Alabama Mitchell Cancer Institute, UNITED STATES

## Abstract

Tumor-associated macrophages (TAMs) play a role in tumor angiogenesis and are recruited into the tumor microenvironment (TME) by secreted chemokines, including Monocyte Chemoattractant Protein-1 (MCP-1/CCL2). Angiogenesis is required to sustain proliferation and enable metastasis of breast cancer (BCa) cells. Understanding the underlying mechanisms of TAM recruitment would allow for the identification of desperately needed novel drug targets. Sushi Domain Containing 2 (SUSD2), a transmembrane protein on BCa cells, was previously shown to promote tumor angiogenesis in a murine model. To identify the role of SUSD2 in angiogenesis, 175 human breast tumors were surveyed by immunohistochemical analysis for the presence of SUSD2 and macrophages. Tumors with high levels of SUSD2 staining contained 2-fold more TAMs, mainly of the M2 pro-angiogenic phenotype. An *in vitro* co-culture model system was developed by differentiating SC monocytes into SC M0 macrophages. A 2-fold increase in polarized M2 macrophages was observed when M0 macrophages were incubated with *SUSD2*-expressing BCa cells compared to cancer cells that do not contain SUSD2. Since MCP-1 is known to recruit macrophages, levels of MCP-1 were compared between *SUSD2*-expressing MDA-MB-231 and MBA-MB-231-vector control cell lines. *MCP-1* RNA, intracellular protein and secreted MCP-1 were all significantly increased compared to the vector control. Knockdown of *SUSD2* in SKBR3 resulted in significantly decreased levels of secreted MCP-1. Consistently, increased levels of MCP-1 were observed in *Susd2*-expressing tumors generated from an *in vivo* isogeneic mouse model compared to the vector control tumors. Because SUSD2 recruits macrophages into the TME and promotes M2 polarization, inhibiting the function of SUSD2 may be an effective therapy for breast cancer patients.

## Introduction

Breast cancers (BCa) are composed of malignant and non-malignant cells, which constitute the tumor microenvironment (TME). Intercellular communication in the TME is regulated by a dynamic network of secreted cytokines, chemokines, growth factors and matrix-remodeling enzymes [1]. Cancer cells manipulate the surrounding non-malignant cells of the TME to secrete tumor-promoting factors that create a more hospitable environment necessary for tumor proliferation, invasion and metastasis [2]. Through this manipulation, tumor cells initiate feedback loops [3], such as the recruitment of myeloid derived macrophages into the TME through the production and release of chemotactic factors [4,5]. Tumor-associated macrophages (TAMs) are highly plastic cells that readily respond to and are reprogrammed by signals found within the TME. Chemotactic factors recruit TAMs to tumors where they are an important source of cytokines and proteases for the promotion of tumor angiogenesis, invasion, immune evasion, metastasis and inhibition of apoptosis [1,3,6,7]. TAMs enhance angiogenesis and increase microvessel density in tumors through the release of growth factors, cytokines and chemokines into the TME [8]. Clinically, there is a strong relationship between TAMs and decreased relapse free survival as well as reduced overall survival of BCa patients [9]. The plasticity of TAMs allows functionally distinct phenotypic changes to occur depending on the chemokine profile to which they are exposed [10]. The M1 classically activated phenotype is often associated with a pro-inflammatory response. The alternatively activated M2 phenotype is associated with anti-inflammatory properties and is involved in angiogenesis, metastasis and the creation of immunosuppressive T-cells [11,12].

Monocyte Chemoattractant Protein-1 (MCP-1/CCL2) is a well-known TAM chemoattractant widely expressed in tumors, including breast, bladder, ovarian and cervical cancers [13]. Clinical evidence suggests that the release of chemokines, such as MCP-1, mediates the migration of monocytes from the blood circulation to breast tumors where they become active macrophages, thus contributing to BCa progression [14,15]. High levels of MCP-1 in breast tumors are associated with early relapse and poor prognosis of patients [16]. MCP-1 is a driver of M2 differentiation of macrophages in the TME, further enhancing its angiogenic and immunosuppressive properties [11,17]. Subsequently, patient tumors containing high levels of TAMs or an abundance of macrophage growth factors in the TME have increased microvessel density and poor overall survival [5,13,14,18].

Angiogenesis has long been recognized as an underlying promoter of BCa progression; however, our understanding of intra-tumoral signaling complexity with the surrounding TME remains limited. Physiologically, angiogenesis is tightly regulated by a delicate balance of anti-angiogenic and pro-angiogenic factors [19]. Tumor cells disrupt angiogenic homeostasis through altered gene expression of secreted cytokines and growth factors [20]. Up-regulation of these secreted factors by the tumor cells promotes infiltration of stromal and vascular cells into the TME, which promotes vascular neogenesis and further enhances angiogenesis within the tumor [1].

The ability of tumor cells to initiate angiogenesis is essential for metastatic spread, making the process an ideal therapeutic target [21,22]. To identify novel targets for BCa, a cDNA library enriched with genes encoding membrane and secreted proteins that are highly expressed in cancer with minimal expression in normal essential tissues was generated [23]. From this cDNA library, we identified Sushi Domain Containing 2 (SUSD2), a type I transmembrane protein that localizes to the cell surface. *SUSD2* was highly expressed in BCa but showed a restricted expression pattern in normal tissues [24]. Using a syngeneic mouse model, we observed accelerated tumor formation, decreased survival of mice and increased angiogenesis in *Susd2*-expressing tumors, suggesting that SUSD2 may play a role in tumor neovascularization [24].

To define a mechanism by which SUSD2 affects angiogenesis, MCP-1 levels were analyzed in patient tumor samples, tumors from an *in vivo* mouse model and several breast cancer cell lines. Consistently, increased intracellular, as well as secreted MCP-1, was associated with high levels of SUSD2 both in culture and *in vivo* studies. Also, increased presence of MCP-1 in the TME was correlated with increased M2 TAMs, thus indicating that the changes in TME initiated by *SUSD2* expression in BCa contribute to the increase in pathological angiogenesis of these tumors.

## Materials and methods

### Patient breast cancer samples

One hundred seventy-five BCa tumor samples were collected at the time of surgery from women newly diagnosed with BCa at Sanford Health, Sioux Falls, SD as previously described [25]. The inclusion criteria for cases were women more than 30 years of age that were newly diagnosed with breast cancer (any type) at Sanford Health. Case subjects were excluded only if they had a previous history of cancer of any kind. All patients provided written informed consent, and the Sanford Health IRB approved the study protocol. Tumor samples from 175 patients with breast cancer were collected from October 8, 2009, to April 17, 2012.

### Cell lines

Stable MDA-MB-231 (ATCC), SKBR3 (ATCC) and 66CL4 [26] cell lines were generated as previously described, and the presence of SUSD2/Susd2 in all stable cell lines was verified by western immunoblot analysis, flow cytometry and immunohistochemical analysis [24]. Human MDA-MB-231 and mouse 66CL4 cell lines do not express *SUSD2/Susd2*. Stable expressing *SUSD2/Susd2* and vector control cell lines were designated MDA-MB-231-SUSD2, 66CL4-Susd2, MDA-MB-231-vector and 66CL4-vector, respectively. The SKBR3 cell line endogenously expresses *SUSD2*. Two SKBR3 SUSD2 knockdown (KD) clones, designated KD1-4 and KD4-4, and SKBR3 non-targeting (NT) cell lines were generated. SC monocytes (CRL-9855) were used as a source of macrophages for *in vitro* assays.

### Tissue culture conditions

Cell lines were cultured in DMEM supplemented with 10% fetal bovine serum (Atlanta Biologicals, Flowery Branch, GA) and 600 μg/mL G418 for MDA-MB-231 and 66CL4 stable cell lines or 0.8 μg/mL puromycin for the stable SKBR3 cell line. Primary Human Umbilical Vein Endothelial Cells (HUVEC) (ATCC PCS-100-013, Manassas, VA) were maintained in Vascular Cell Basal Medium (ATCC PC-100-030) enhanced with the ‘Endothelial Cell Growth Kit-VEGF’ (ATCC PCS-100-041). All cell lines were cultured at 37°C with 5% CO_2_ in a humidified atmosphere. All cell lines were authenticated and tested negatively for mycoplasma.

### Immunohistochemical staining

Blocks of formalin-fixed, paraffin-embedded mouse and human breast tissue were prepared for immunohistochemical (IHC) analysis. Tissues were sectioned at 5 μm. Optimization and staining of all antibodies was performed on the BenchMark XT automated slide staining system (Ventana Medical Systems, Inc., Tucson, AZ). The Ventana iView DAB detection kit was the chromogen, and slides were counterstained with hematoxylin. Omission of primary antibody served as the negative control. Antibodies used: anti-SUSD2 (Prestige Antibodies, St. Louis, MO), anti-HER2/ErbB2 (Cell Signaling, Danvers, MA), anti-MCP-1 (MyBioSource, San Diego, CA), anti-F4/80 (AbD SeroTech, Raleigh, NC), anti-CD68 (Cell Marque, Rocklin, CA), anti-CD163 and anti-MHCII (Thermo Fisher Scientific, Waltham, MA).

### Scoring of immunohistochemistry staining

A trained pathologist (C.T.S.) scored the human tumors. *HER2* analysis was performed using standard clinical laboratory protocols [27]. SUSD2 staining was scored as described previously by Kwon et al [28]. Extensiveness was scored as follows: 0 (<5% immunoreactive), 1 (5%-32% immunoreactive), 2 (33%-66% immunoreactive), 3 (>66% immunoreactive). Intensity of staining was scored with the following scale: 0 (negative), 1 (weak), 2 (moderate), 3 (strong). A combined score was calculated by adding the scores for extensiveness (scored 0–3) and intensity (scored 0–3). The combined scores were ranked by quartiles into composite scores: 0, +1 (1–2 combined score), +2 (3–4 combined score) and +3 (5–6 combined score). When a stained tumor section contained both *in situ* and invasive components, areas were scored separately, generating a composite score for each component. CD68 IHC staining was scored by counting macrophages per high power field (hpf) in three hot spots per tumor at 200x. A hot spot is a pathological analysis of an area of increased staining density [29]. First, the entirety of the tumor is evaluated on low power, identifying clusters of dense staining. Second, individual macrophages are then counted at a higher power (200x) in the identified hot spot.

### Mouse model

All animal experiments were approved by the IACUC at Sanford Research, Sioux Falls, SD. Sanford Research has an Animal Welfare Assurance on file with the Office of Laboratory Animal Welfare (A-4568-01) and is a licensed research facility under the authority of the United Sates Department of Agriculture (46-R-0011). Seven-week-old female Balb/c mice (Charles River Laboratories) were subcutaneously injected with 1x10^5^ syngeneic 66CL4-Susd2 or vector cells in 100 μl serum-free DMEM into the lower right mammary fat pad region. Tumor size was measured every 2–3 days once a palpable tumor had formed. Tumor volume was calculated with the following formula: volume = π/6 x (short diameter)^2^ x (long diameter) [30]. Mice were sacrificed when moribund or tumor volume reached 1500 mm^3^ [24]. Euthanasia was performed by asphyxiation of inhaled 100% CO_2_. Tumors were removed during the necropsy, fixed in 10% formalin and paraffin-embedded.

### RT-qPCR

*MCP-1* expression was determined and verified by quantitative reverse transcriptase PCR (RT-qPCR). Total RNA was extracted according to the manufacturer instructions (Stratagene). Reverse transcription was carried out using total RNA (5μg) from the indicated cell line as a template. Primers for MCP-1 (forward 5’-CCAGTCACCTGCTGTTAT-3’ and reverse 5’-CAATGGTCTTGAAGATCACA-3’) and GAPDH (forward 5’-AGCCACATCGCTC-AGACAC-3’ and reverse 5’-GCCCAATACGACCAAATCC-3’) were synthesized by Integrated DNA Technologies (Coralville, IA).

### Western immunoblot analysis

Western immunoblot analysis was performed as previously described [24]. Equal loading was verified using anti-glyceraldehyde-3-phosphate dehydrogenase (GAPDH) antibody. Primary antibodies include: monoclonal mouse anti-MCP-1 (R&D Systems, Minneapolis, MN) and monoclonal mouse anti-GAPDH (GeneTex, Irvine, CA).

### Monocyte differentiation and macrophage polarization

SC monocyte cells were plated at a density of 7.5x10^5^ cells/mL in DMEM supplemented with 10% fetal bovine serum and 10 ng/mL phorbol 12-myristate 13-acetate (PMA). Cells were incubated for 48 hours at 37°C with 5% CO_2_. After 48 hours, media with PMA was removed and cells were washed with PBS. Cells were then incubated for an additional 24 hours in DMEM supplemented with 10% fetal bovine serum to rest the cells before experiments were performed. Without further stimulation, these cells were designated SC derived M0 macrophages (SC M0). Macrophages were polarized to the M1 phenotype using 20 ng/mL IFN-γ for 24 hours and to the M2 phenotype using 20 ng/mL each of IL-4 and IL-13 for 24 hours.

### Macrophage co-culture assay

For co-culture experiments, 2x10^6^ SC monocytes were differentiated to SC macrophages using phorbol 12-myristate 13-acetate (PMA). 10 ng/mL PMA was added to SC monocytes for 48 hours. Following PMA stimulation, SC M0 macrophages were rested in complete medium without PMA for 24 hours before co-culture. 1x10^6^ MDA-MB-231-SUSD2 or MDA-MB-231-vector cells were co-cultured with SC M0 macrophages for 24 hours. Cell co-cultures were harvested by gentle scraping, and cells were pelleted, fixed in 10% formalin, and sectioned for IHC staining. Entire slides were imaged using Aperio VERSA slide scanner. Positive cells were counted using Aperio Image Analysis.

### Luminex assay

MCP-1 levels in cell culture supernatants of MDA-MB-231-SUSD2, MDA-MB-231-vector, SKBR3-NT, SKBR3 KD1-4 and SKBR3 KD4-4 were analyzed with anti-MCP-1-coated magnetic Luminex beads from R&D Systems (Minneapolis, MN) according to manufacturer’s instructions. Briefly, 50 μl of undiluted supernatant was incubated with the beads at room temperature (RT) for 2 hours. After washing, a biotinylated anti-MCP-1 secondary antibody was added to the beads and incubated at RT for 1 hour. The beads were washed again and incubated with Streptavidin-PE for 45 minutes at RT. After a final washing step, the beads were re-suspended and analyzed with a Luminex 100/200 instrument.

### Human umbilical vein endothelial cell (HUVEC) tubule assay

Stable MDA-MB-231-SUSD2, MDA-MB-231-vector, SKBR3 KD1-4, SKBR3 KD4-4 and SKBR3-NT cell lines were incubated at 37°C with serum-free media overnight. Wells of a 24-well plate were coated with 75 μl of Growth Factor Reduced (GFR) Matrigel (BD Biosciences, San Jose, CA) and incubated at 37°C for 30–60 minutes. HUVEC (3 x 10^4^ cells/well) were suspended in 500 μl/well of the conditioned media under study and plated on the Matrigel. Plates were incubated at 37°C in 5% CO_2_ with humidity for 6 hours. After incubation, imaging of five representative fields per well was performed using phase contrast microscopy. The number of branch points was counted using ImageJ software.

### Statistics

Where indicated, student’s t-test was used to compare two groups. One-way ANOVA analysis was used with a Tukey post-test when greater than two groups were being compared. For patient tumors, natural logarithm transformation was used to improve normality and homoscedasticity in residual errors. A cross-sectional study design of BCa tissue samples was used to determine the associations between SUSD2 staining (sum of intensity and extensiveness) and tumor characteristics (SAS version 9.3 software).

## Results

### Immunohistochemical (IHC) analysis of SUSD2 in patient breast tumor samples and mouse mammary gland tumors

Previously, we reported that SUSD2 is present in 82% of a limited number of patient tumors studied [24]. To perform a statistical analysis to correlate SUSD2 staining with BCa subtypes, including Estrogen Receptor (ER)+, *HER2* amplified and triple-negative subtypes, a larger cohort of breast tumors was required. Tumors were collected from 175 women over the age of 30 who had been recently diagnosed with BCa. The pathology reports, including tumor size, *in situ*, invasive, hormone receptor status, *HER2* amplification and lymph node involvement, were obtained. Because clinical *HER2* amplification testing is only performed for invasive BCa tumors, we performed HER2 staining using an anti-HER2 antibody on 42 *in situ* tumors in order to complete the data set. A trained pathologist scored the IHC stained tissue sections. Eighteen of 42 *in situ* tumors demonstrated *HER2* amplification and were categorized accordingly. To determine the status of SUSD2 in the patient samples, the 175 tumors were stained by IHC using an anti-SUSD2 antibody. We have previously validated the specificity of the anti-SUSD2 antibody used for IHC analysis [24], and S1 Fig demonstrates the staining pattern of SUSD2 in cell lines. The tumor samples were scored for intensity and extensiveness of staining (0, 1+, 2+, 3+, see Materials and Methods). Consistent with our previous analysis [24], 80% of the 175 evaluated breast tumors had moderate to strong staining for SUSD2 (2+/ 3+ composite score) (Table 1). Additionally, 91% of HER2 amplified tumors had moderate to strong staining of SUSD2, which was significantly higher than the other subtypes (p<0.03). No association between SUSD2 staining and lymph node involvement, tumor size, patient age, patient BMI, patient smoking status or family history was observed (data not shown).

**Table 1:** Summary of SUSD2 staining of 175 breast cancer patient samples.

175 Tumors Total	No SUSD2 Staining 0	Weak SUSD2 Staining 1+	Moderate SUSD2 Staining 2+	Strong SUSD2 Staining 3+	^a^Moderate + Strong
**Invasive** (n = 133)	8% (11)	13% (17)	41% (55)	38% (50)	79%
***in situ*** (n = 42)	7% (3)	12% (5)	48% (20)	33% (14)	81%
**ER+** (n = 145)	9% (13)	14% (20)	42% (61)	35% (51)	77%
**PR+** (n = 123)	9% (11)	14% (17)	45% (55)	32% (40)	77%
**HER-2+** (n = 32)	3% (1)	6% (2)	38% (12)	53% (17)	91%
**Triple Negative** (n = 15)	7% (1)	13% (2)	20% (3)	60% (9)	80%
**LN Involvement** (n = 45)	2% (1)	18% (8)	38% (17)	42% (19)	80%

IHC analysis was performed using an anti-SUSD2 antibody on 175 patient breast tumors representing the following subtypes: invasive, *in situ*, ER positive, PR positive, HER-2 amplification, triple negative and lymph node (LN) involvement. The number of tumors is each group is indicated by parentheses. Tumors were placed into groups according to SUSD2 staining score, 0, 1+, 2+ and 3+. The percentage of tumors stained is indicated.

^a^The percentage of tumors with moderate (2+) SUSD2 staining plus strong (3+) SUSD2 staining.

### Increased levels of tumor-associated macrophages are observed in *SUSD2-*expressing cells

**Fig 1:**
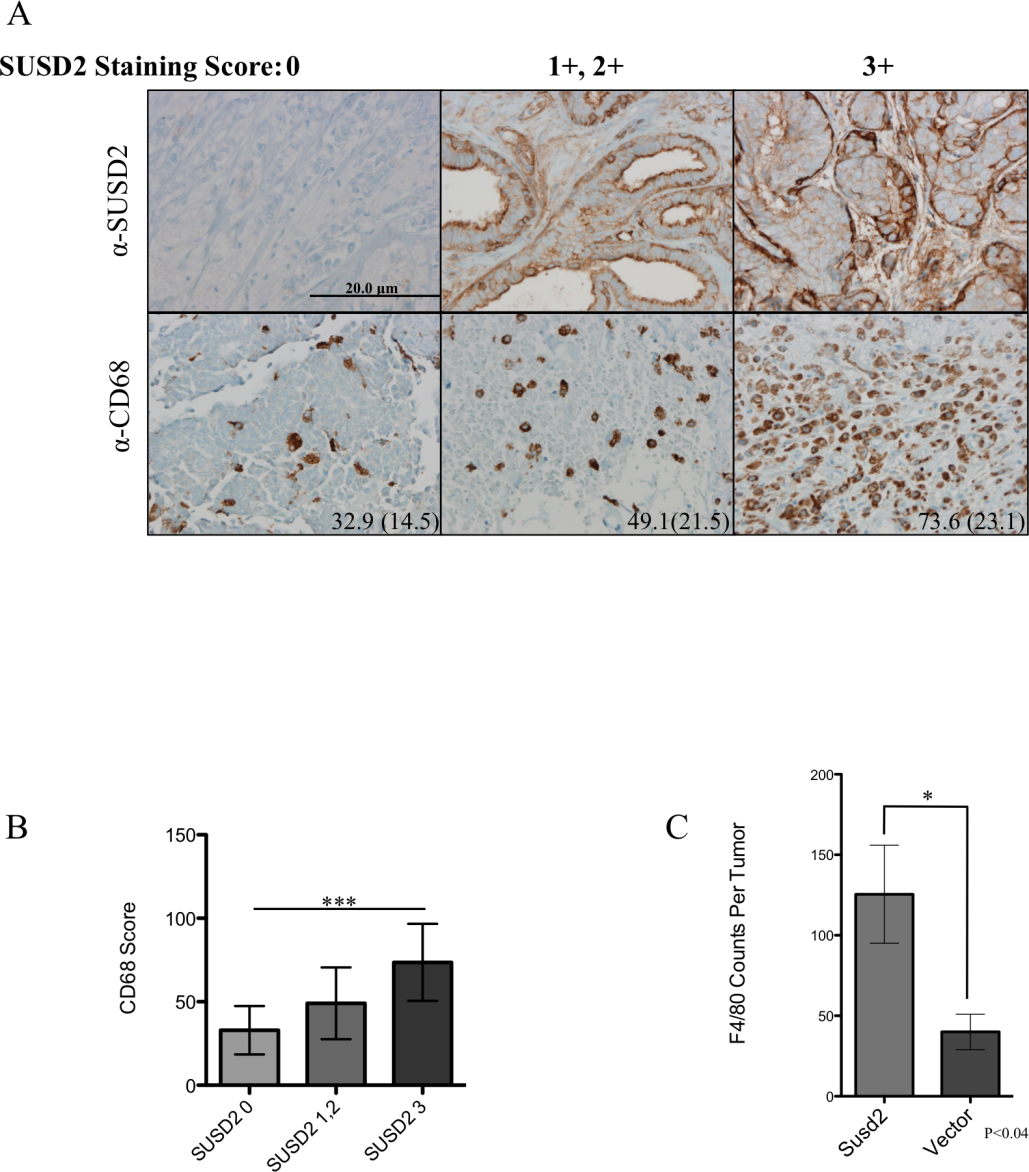
SUSD2 is associated with increased macrophages in breast tumors. (A) Tissue sections from women undergoing surgical treatment for BCa were analyzed by immunohistochemical analysis using anti-SUSD2 and anti-CD68 (macrophage marker) antibodies. Positive staining is indicated by the brown color. Cells were counterstained blue with hematoxylin. CD68 scores were obtained by counting the number of positive staining cells per high power field (hpf) in three hot spots throughout the tumor. Representative images of each sample are shown. Left, middle and right columns are sections from tumors with a SUSD2-staining score of 0, 1+/2+, and 3+, respectively. The average score with standard deviation in parentheses is located in the bottom right corner of the image for CD68 staining. Images were taken at 200× magnification. (B) Association between CD68 score and SUSD2 staining in patient tumors. Data are represented as mean. Error bars indicate standard deviation (*** p<0.0001). (C) The total number of macrophages per tumor section of 66CL4 mouse tumors stained with anti-F4/80 activated macrophage marker.

To determine the extent of macrophage infiltration into the tumors, human tumor sections were stained using anti-CD68 (macrophage marker) antibody. Staining was associated with the presence of SUSD2 (1A). Tumors were placed into three groups according to the composite SUSD2 staining scores (group 1, 0; group 2, 1+/2+; group 3, 3+). Samples were evaluated for the presence of TAMs by counting the number of CD68 positive cells in three hot spot high power fields (hpf) per tumor. A hot spot is a pathological analysis of an area of high density staining [29]. The entirety of the tumor is evaluated on low power allowing for clusters of dense brown anti-CD68 staining to be identified. That area is then examined using a higher power (200x), and the individual macrophages are counted in one field at 200x magnification. Shown in Fig 1B, the average number of macrophages per hpf in the tumors increased with SUSD2 staining: 32.9, 49.1 and 73.6, which corresponds with SUSD2 scores of 0, 1+/2+, and 3+, respectively. Using a one-way AVOVA, SUSD2 staining was strongly associated with CD68 macrophage staining (p<0.001). This result suggests that SUSD2 modulates the recruitment of macrophages to the TME.

After observing increased numbers of macrophages in patient tumors with strong SUSD2 staining, we analyzed tumors isolated from a previously studied isogeneic 66CL4 BALB/c mouse model [24] to determine if the mouse model is consistent with the results generated using human samples. The 4T1 mammary carcinoma cell line was derived from a spontaneously arising mammary tumor in a Balb/c mouse [26]. Because the 4T1 cell line is very aggressive, we utilized a 4T1 sibling cell line, 66CL4 [26]. Both 4T1 and 66CL4 cell lines have been classified as triple negative (estrogen receptor negative, progesterone receptor negative and *HER-2* amplification negative) [31]. Since 66CL4 wild-type cells do not endogenously express *Susd2*, stable *Susd2*-expressing and vector control cell lines were established and extensively characterized by flow cytometry, western immunoblot analysis and IHC, as published in Watson, et al., 2013 [24]. The cell lines were subcutaneously injected into the lower right mammary fat pad region of 7-week old BALB/c mice according to the Sanford Research IACUC approved protocol. After mice were sacrificed, tumors were removed and paraffin-embedded [24]. Macrophage recruitment to the area of the 66CL4-Susd2 and vector control tumors was evaluated by IHC analysis using an anti-F4/80 macrophage marker. Consistent with the results of the patient samples, 66CL4-Susd2 tumor sections contained >2-fold more macrophages compared to vector control (Fig 1C).

### Amount of M2 polarized macrophages increases with SUSD2

**Fig 2:**
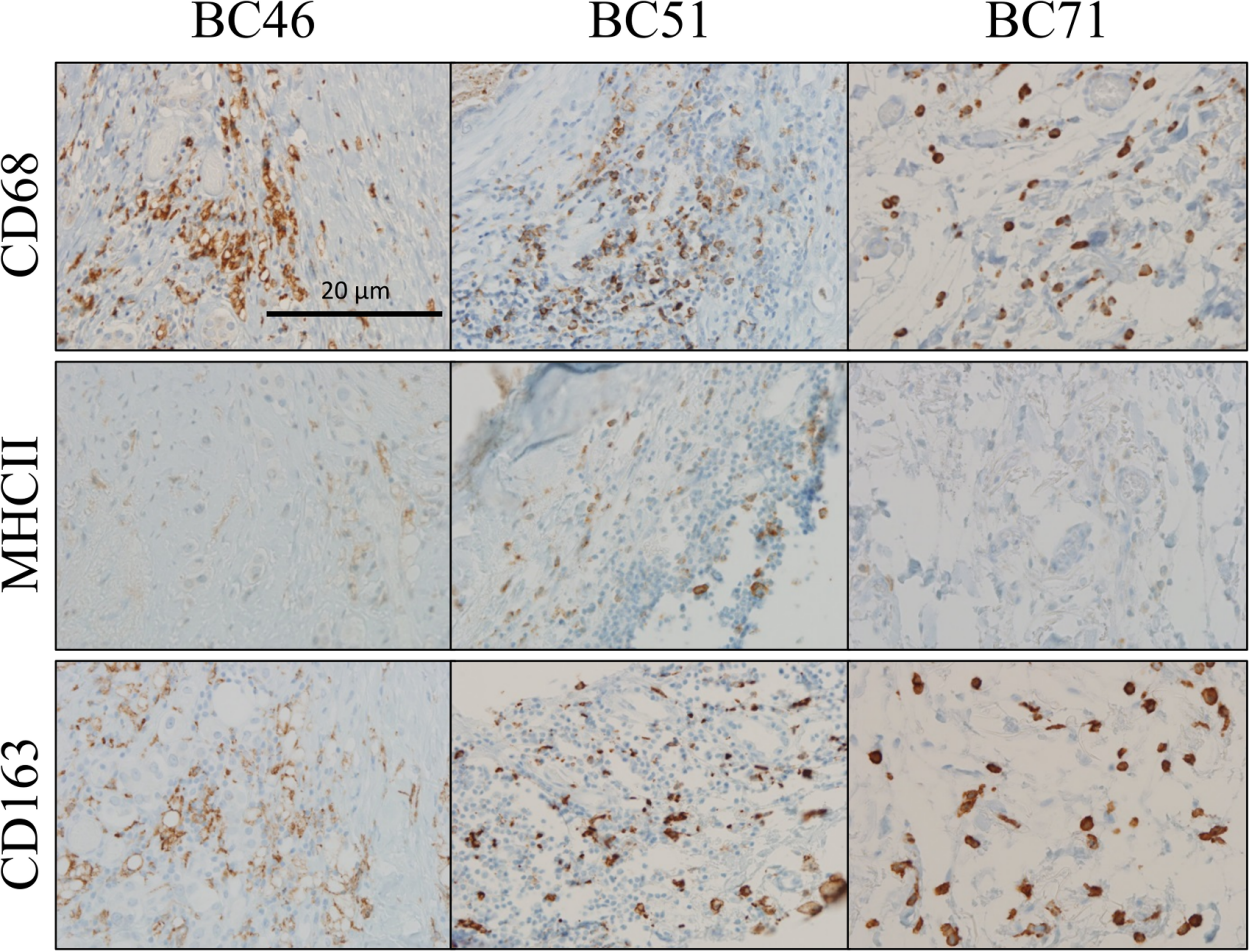
Immunohistochemical analysis of M1 and M2 macrophage polarization in breast tumors. Tumor sections with strong (3+) SUSD2 staining were analyzed from ten breast cancer patients (representative tumors BC46, BC51, and BC71 are shown). The patient tumors were immunostained using anti-MHCII (M1 marker) and anti-CD163 (M2 marker) antibodies. The brown color indicates positive staining, and the cells were counterstained blue with hematoxylin. Images were taken at 200× magnification. Representative images are shown. Similar areas of each of the tumors were imaged in all three serial sections.

To determine the polarity of the TAMs in the BCa tumors, the 3+ SUSD2 staining patient samples were evaluated for M1 or M2 polarity using anti-MHCII or anti-CD163 antibodies, respectively. Areas of TAMs indicated by CD68 staining (Fig 1A) were compared in similar areas of serially sectioned tumors. In tumors with strong SUSD2 staining, the increased numbers of TAMs were predominantly of the M2 phenotype, identified by the anti-CD163 staining. Very few of the TAMs were M1 phenotype, as seen by the weak MHCII staining (Fig 2).

**Fig 3:**
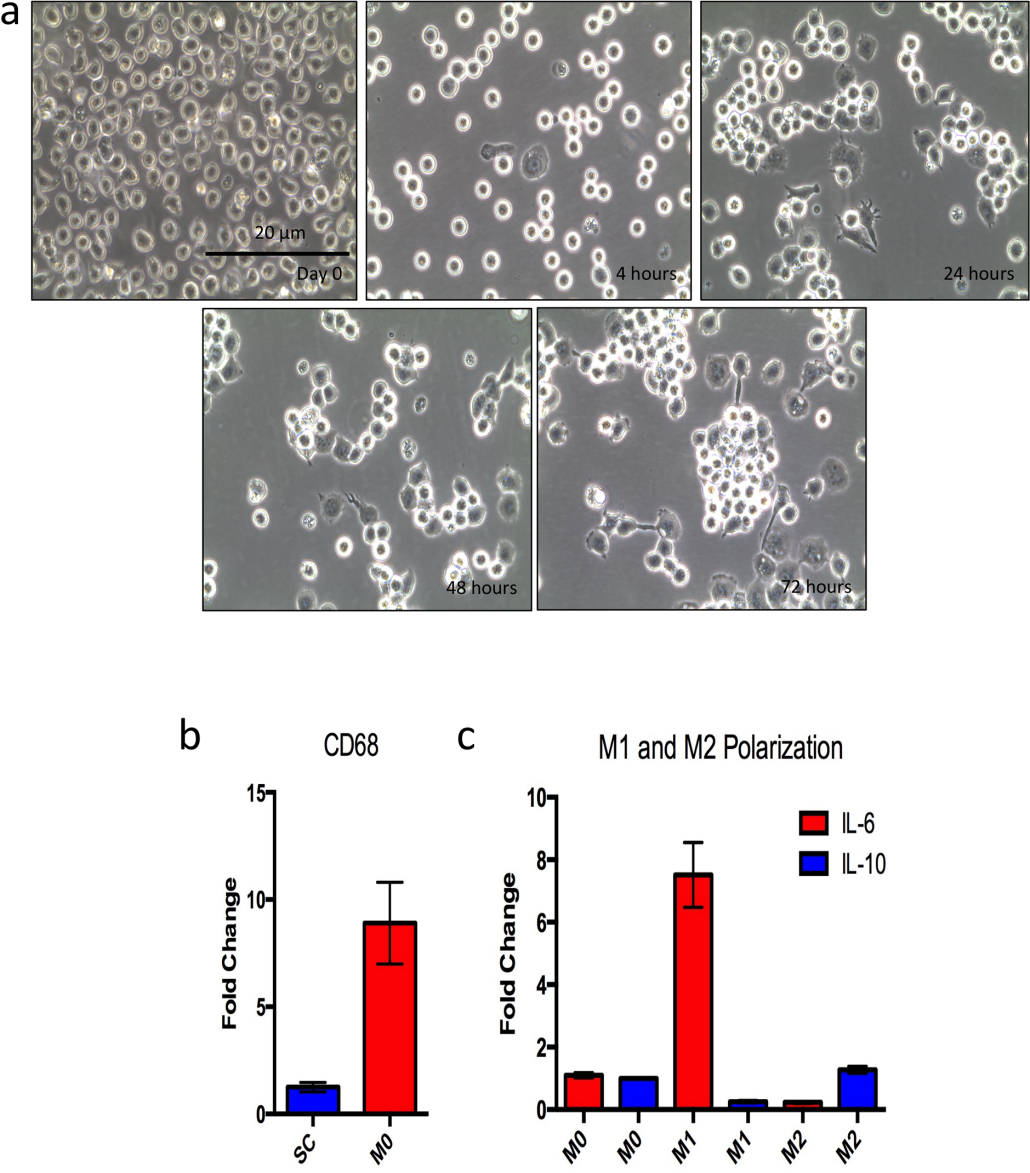
Monocyte to macrophage differentiation and polarization. SC monocytes were stimulated for 48 hours with 10 ng/mL PMA to achieve differentiation to macrophages. Macrophages were rested for 24 hours in DMEM 10% FBS with no PMA before experiments. A) Morphological changes observed over the differentiation timeline; day 0 shows monocytes before PMA treatment. The subsequent images show time after PMA stimulation. B) qPCR analysis of *CD68* gene expression in SC derived M0 macrophages compared to SC monocytes. RNA was isolated from SC monocytes and SC M0 macrophages, and total RNA was used as a template for cDNA synthesis. Real time qPCR analysis was performed, and *CD68* expression was normalized to GAPDH. Fold change was set relative to SC monocyte CD68 expression. C) M1 and M2 polarization of SC M0 macrophages. M1 macrophages were differentiated using 20 ng/mL IFN-γ for 24 hours. M2 macrophages were generated using 20 ng/mL each of IL-4 and IL-13 for 24 hours. qPCR was performed to analyze expression of M1 and M2 genes. IL-6 was used as a classical M1 marker, while IL-10 was used as a classical M2 marker.

An *in vitro* co-culture assay was developed to support the findings in patient tumor sections that SUSD2 skews macrophages towards an M2, pro-tumor phenotype. The SC monocyte cell line was used as a source of macrophages. These cells were isolated from peripheral blood and immortalized *in vitro*. Since they were not derived from cancer cells, they may represent a more physiological response compared to the monocyte cell lines currently available. Yamamoto et al found that SC monocytes more closely resembled peripheral blood monocytes in their response to pyrogens compared to other monocyte cell lines including THP-1 [32]. Before performing co-culture experiments, we examined the differentiation characteristics of this cell line. SC monocytes were differentiated to SC macrophages using phorbol 12-myristate 13-acetate (PMA). After PMA treatment, we refer to the SC cells as SC M0. The SC M0 cells underwent phenotypic and morphologic changes that are associated with macrophage differentiation. SC monocytes are a suspension cell line. However, after 4 hours of PMA stimulation, the SC M0 cells started to adhere to the culture plate and continued to adhere after PMA was removed from the growth media (Fig 3A). The morphology of the SC M0 cells changed over time from a rounded shape to a more flattened, spread-out shape with filopodia. This morphological transition is characteristic of monocyte to macrophage differentiation. Additionally, the SC M0 cells could be cultured for over 2 weeks and passaged 2–3 times before cell death (data not shown), which is consistent with monocyte to macrophage differentiation in other models [33].

To further characterize the monocyte to macrophage differentiation, we analyzed gene expression of CD68 (macrophage marker) in SC monocyte and SC M0 cells using RT-qPCR. Compared to SC monocyte cells, SC M0 cells showed an 8-fold increase in *CD68* expression (Fig 3B). To determine whether the SC M0 cells retained the ability of polarizing to either M1 or M2 macrophages, the SC M0 cells were incubated with either IFN-γ to generate M1 macrophages or both IL-4 and IL-13 to generate M2 macrophages. We observed an increase in *IL-6* gene expression (characteristic of M1 macrophages) after M1 differentiation and a decrease in *IL-6* gene expression following M2 differentiation (Fig 3C). Consistently, we observed the reciprocal response for IL-10 (M2 macrophage). M1 macrophages showed decrease in *IL-10*, while M2 macrophages showed an increase in *IL-10* gene expression (Fig 3C). This result indicated that the SC M0 cells still have the capacity to be polarized to M1 or M2 macrophages.

**Fig 4:**
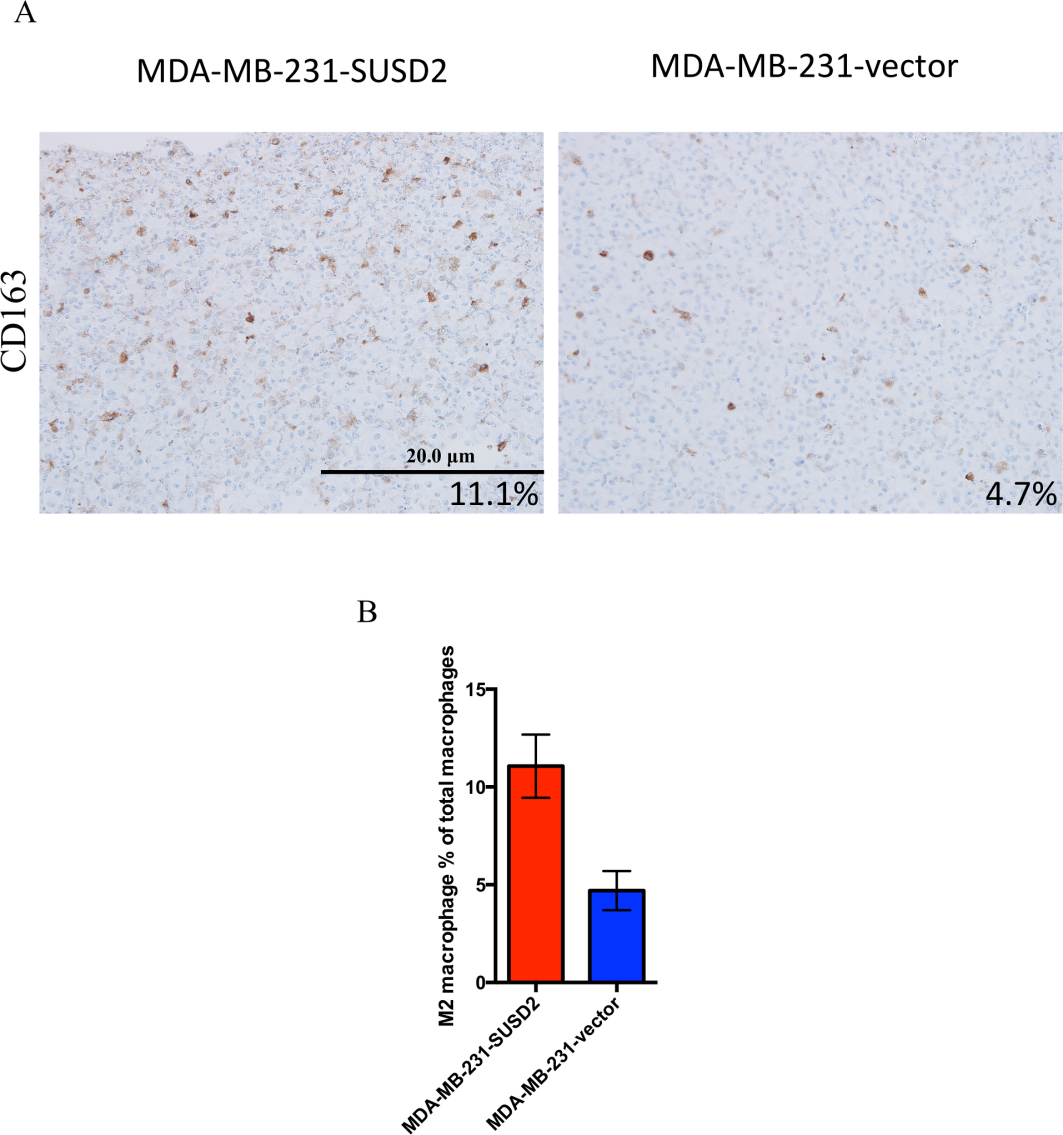
M2 macrophage number increases after co-culture with MDA-MB-231-SUSD2. Macrophages co-cultured with MDA-MB-231-SUSD2 and MDA-MB-231-vector are shown. SC monocyte cell line was differentiated with PMA to generate SC M0 macrophages. MDA-MB-231-SUSD2 or vector cells were then added and co-cultured with SC M0 macrophages for 24 hours. A) Cells were collected, pelleted, fixed, paraffin-embedded and sectioned for IHC staining of CD68 and CD163. Cells were counterstained with hematoxylin. Images were taken at 200x. B) M2 macrophage percentage was determined by dividing CD163 (M2 macrophage marker) positive cells by CD68 (pan macrophage marker) positive cells in serial sections for three fields per condition. ImageJ software was used to count positively stained cells. Quantification is from a representative experiment of two biological replicates.

To determine whether SUSD2 affects macrophage polarization, SC M0 macrophages were co-cultured directly with MDA-MB-231-SUSD2 or -vector cells to allow for contact between cells. After 24 hours, the co-cultures were pelleted, fixed and paraffin-embedded for IHC analysis. Serial sections were stained with either anti-CD68 antibody, a macrophage marker, or anti-CD163 antibody to identify only the M2 activated macrophages. Cells were counterstained with hematoxylin, which is blue, and brown stain indicates positive staining (Fig 4). Representative images of CD163 staining are shown in Fig 4, and the blue staining corresponds to both cancer cells and non-M2 macrophages. The entire sections were imaged using the Aperio VERSA slide scanner and counted using Aperio image analysis software. CD163 positive cells (Fig 4) were divided by the number of CD68 positive cells (data not shown) to obtain percentages of M2 macrophages (CD163) within the total population of macrophages (CD68). Macrophages co-cultured with MDA-MB-231-SUSD2 showed a greater than 2-fold more CD163 positive macrophages compared to macrophages co-cultured with MDA-MB-231-vector cells (Fig 4).

### Increased levels of MCP-1 are observed in *SUSD2-*expressing cells

**Fig 5:**
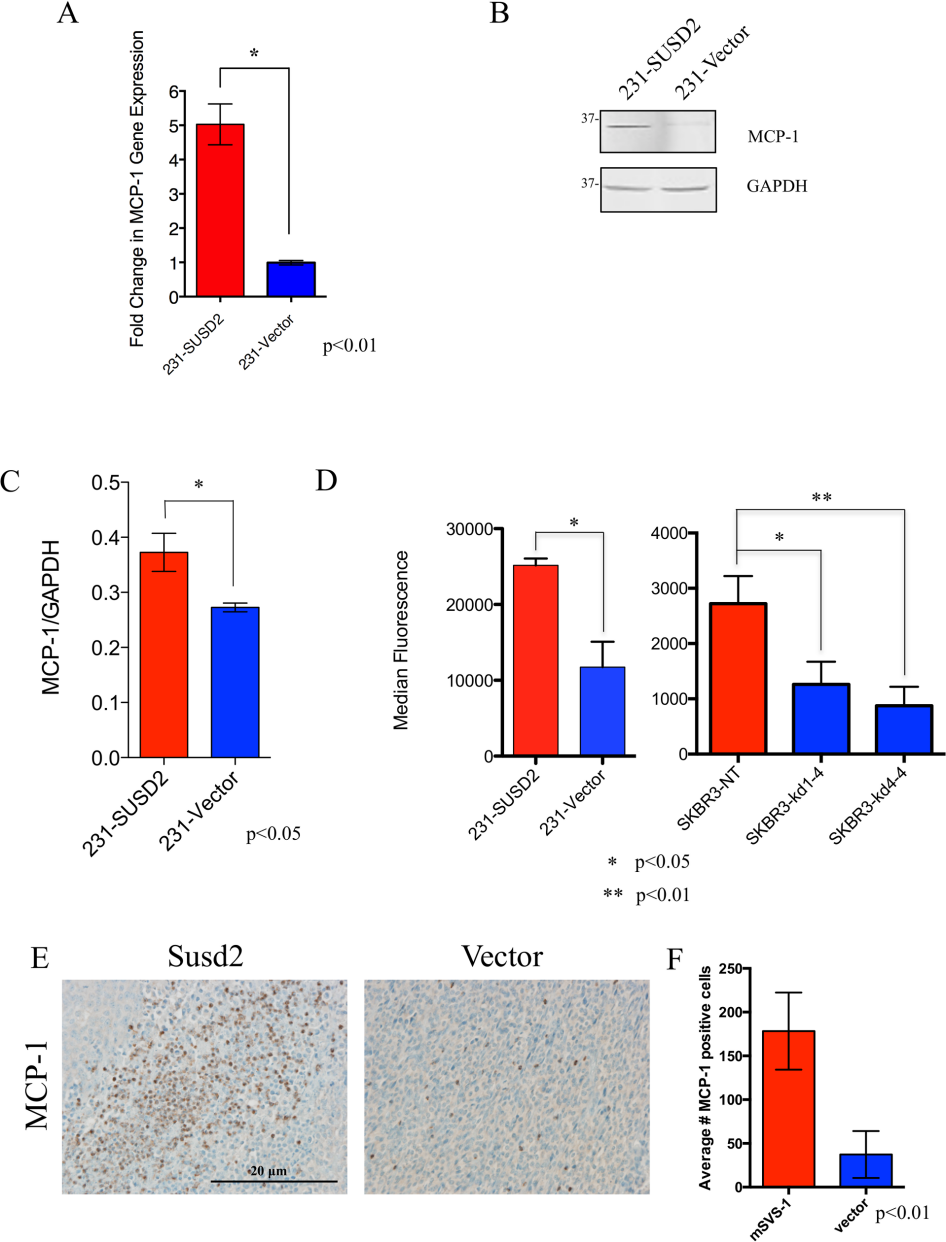
Cells that express *SUSD2* produce increased levels of MCP-1. (A) Effect of SUSD2 on *MCP-1* RNA levels. RTqPCR analysis of *MCP-*1 using cDNA isolated from MDA-MB-231-SUSD2 and vector cell lines was performed. Results were normalized to *GAPDH* and were reported as fold-change over vector. (B) Western immunoblot analysis of MCP-1 levels. Protein lysates generated from MDA-MB-231-SUSD2 and vector cell lines were used to perform western immunoblot analysis using an anti-MCP-1 antibody. GAPDH served as a loading control. (C) Protein bands from panel b were quantified by densitometry, and values are indicated relative to those of GAPDH. Blots were performed in triplicate, and a student’s t-test was used to test for significance. Error bars indicate SEM. (D) Luminex analysis of MCP-1 in cell culture supernatants. MCP-1 levels present in the supernatant of MDA-MB-231-SUSD2, MDA-MB-231-vector, SKBR3-NT, SKBR3 KD1-4 and SKBR3 KD4-4 were measured with an anti-MCP-1 Luminex assay. Results show data from two independent experiments measuring a minimum of 50 beads per assay. (E) IHC staining of MCP-1 in 66CL4-Susd2 (left) and 66CL4-vector (right) tumors excised from syngeneic BALB/c mice. Representative images were taken at 200x magnification. (F) Quantification of MCP-1 IHC in BALB/c mouse tumors. Three representative MCP-1 IHC images were quantified from both 66CL4-Susd2 and 66CL4-vector tumors.

Since MCP-1 is a known recruiter of TAMs and is associated with an M2 macrophage phenotype, the effect of SUSD2 on *MCP-1* expression was examined [16]. RT-qPCR was performed using cDNA generated from MDA-MB-231-SUSD2 and vector control cell lines. After normalizing the data to *GAPDH*, *MCP-1* expression was 5-fold higher in MDA-MB-231-SUSD2 cells compared to the vector control cell line (Fig 5A). To confirm that *MCP-1* RNA levels correlated to protein levels within the cell, western immunoblot analysis was performed using protein lysates harvested from MDA-MB-231-SUSD2 and vector cell lines. Membranes were probed with an anti-MCP-1 antibody. GAPDH was used as a loading control (Fig 5B). Compared to the vector, MDA-MB-231-SUSD2 cells had significantly higher levels of MCP-1 (Fig 5C), which is consistent with corresponding RNA levels.

As MCP-1 is a secreted protein, MCP-1 levels in the supernatant should be higher from cells that express *SUSD2*. Secreted levels of MCP-1 by MDA-MB-231-SUSD2 versus -vector were measured by Luminex assays. Anti-MCP-1-coated magnetic Luminex beads were incubated with cell culture supernatants and analyzed for presence of MCP-1. The culture supernatant from MDA-MB-231-SUSD2 cells contained >2-fold more MCP-1 compared to supernatant from the MDA-MB-231-vector control cell line (Fig 5D). To verify that this result is not specific to one cell line, we also examined SKBR3, which endogenously expresses *SUSD2*. Previously we generated two *SUSD2* knock-down cell lines, SKBR3-KD1-4 and SKBR3-KD4-4, and the non-targeting control cell line, SKBR3-NT. SUSD2 levels in the cell lines were extensively characterized by flow cytometry, western immunoblot analysis and IHC, as published in Watson, et al., 2013 [24]. SKBR3-KD1-4 is more of a complete knock-down of SUSD2 compared to the partial SUSD2 knock-down of SKBR-KD4-4 (S1 Fig). Luminex assays were performed to measure secreted levels of MCP-1 in the conditioned media from SKBR3-KD1-4, -KD4-4 and–NT. Consistent with the results from the MDA-MB-231 cell lines, the SKBR3-NT cells, which express *SUSD2*, secreted >2-fold more MCP-1 than the corresponding SUSD2 KD cell lines (Fig 5D).

Tumors derived from the isogeneic 66CL4 BALB/c mouse model [24] were used to determine if MCP-1 was increased in the tumor milieu *in vivo*. IHC analysis using an anti-MCP-1 antibody was performed on 66CL4-Susd2 or vector tumor sections. MCP-1 was more abundant in tumors expressing *Susd2* compared to vector control tumors (Fig 5E and 5F). The consistent increase in levels of MCP-1 and TAMs with corresponding levels of SUSD2 suggests that *SUSD2*-expressing BCa cells may be increasing angiogenesis through secretion of MCP-1, which recruits TAMs to support a pro-angiogenic TME.

### Conditioned media (CM) from *SUSD2*-expressing cells increases HUVEC tubule formation

**Fig 6:**
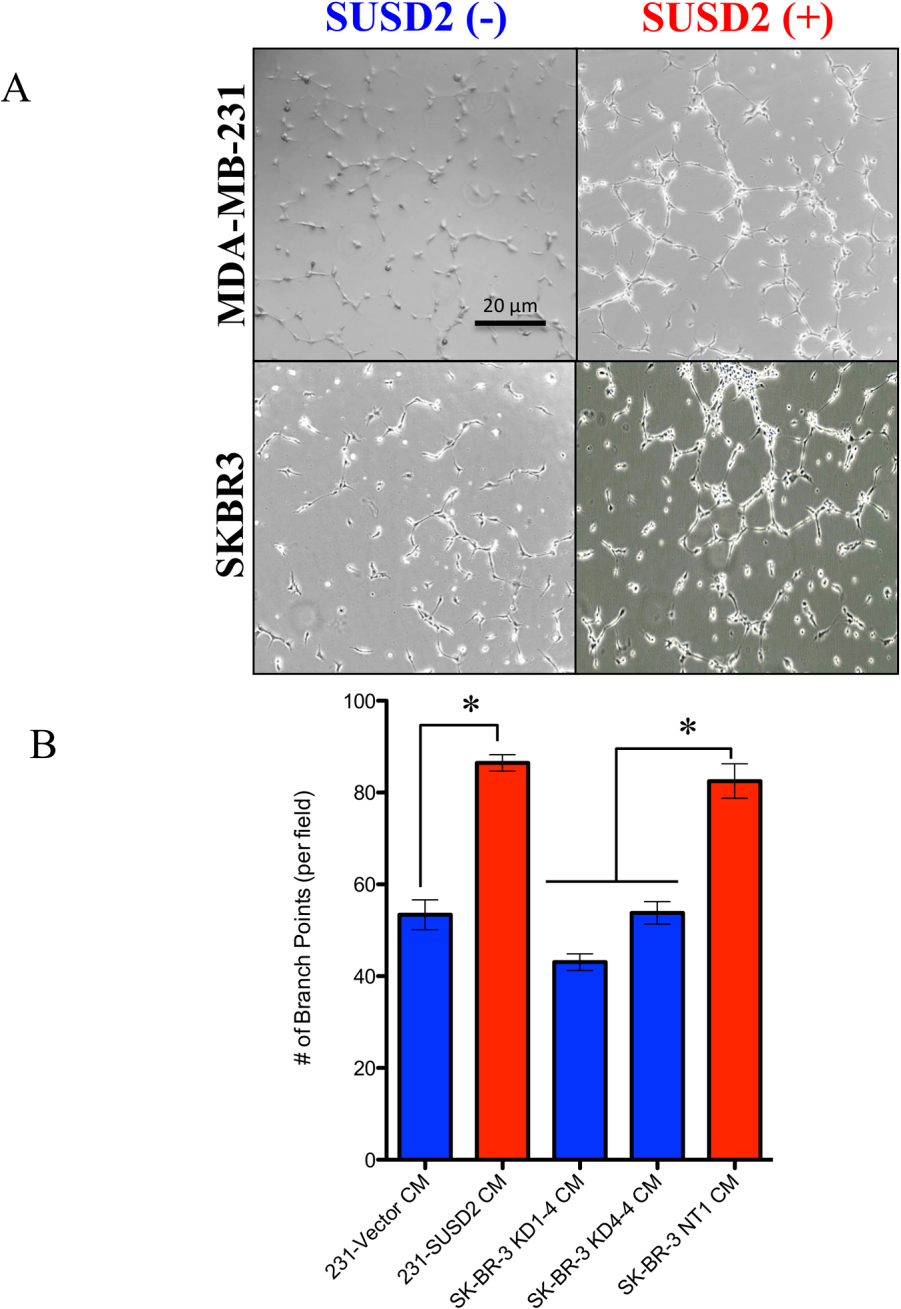
Conditioned media from *SUSD2*-expressing cells increases HUVEC tubule formation. HUVECs were grown on Matrigel coated plates in the presence of conditioned media from MDA-MB-231-SUSD2, MDA-MB-231-vector, SKBR3-NT, SKBR3 KD1-4 or SKBR3 KD4-4 cell lines. (A) Photos using a phase contrast microscope were taken 6 hours after conditioned media was added to the HUVECs. The photos demonstrate the ability of HUVECs to form capillary-like tubules. Images are representative of three independent experiments. The SKBR3 SUSD2(-) cell line shown is SKBR3 KD1-4. (B) Branch points formed within the honeycomb-like pattern were counted and quantified per visual field. Student’s t-test and ANOVA analysis were used to test statistical significance. Error bars indicate standard error of the mean (* p<0.05).

To investigate if the presence of SUSD2 on cancer cells alters the secretion of angiogenic factors, Human Umbilical Vein Endothelial Cell (HUVEC) tubule formation assays were performed (Fig 6A). Endothelial cells form capillary-like structures *in vitro* when plated upon a reconstituted basement membrane in the presence of angiogenic factors [34]. Conditioned media (CM) was collected from MDA-MB-231 and SKBR3 stable cell lines (S1 Fig). HUVECs that were incubated with CM from cell lines producing SUSD2 generated nearly two-fold greater HUVEC branch points compared to HUVECs incubated with CM from cell lines that did not produce SUSD2 (Fig 6B). Additionally, HUVECs incubated with media collected from the complete *SUSD2* knockdown, SKBR3 KD1-4, had fewer branch points than media from the partial knockdown, SKBR3 KD4-4. Because CM from cell lines producing SUSD2 increased tubule formation of HUVECs compared to CM collected from the control cell lines, this result suggests that SUSD2 alters the cytokine profile by increasing the secretion of pro-angiogenic factors.

## Discussion

Angiogenesis is a physiological process in development that is adopted in tumorigenesis to bolster the proliferation of cancer cells. Tumors exploit angiogenesis for metastatic spread by tipping the balance of normal angiogenic homeostasis in favor of new vessel formation through the release of cytokines, chemokines and growth factors into the TME. Previously, our laboratory showed that SUSD2 increases tumorigenesis using an isogeneic mouse model [24]. In the present study, we correlate these findings to BCa patient samples and identify a mechanism by which SUSD2 facilitates macrophage recruitment and angiogenesis in tumors. Using IHC analysis, 175 BCa patient tumors were stained for SUSD2 and macrophages. Approximately 80% of tumors had moderate to strong SUSD2 staining regardless of subtype or tumor characteristics. Interestingly, the presence of SUSD2 was not mediated by hormone receptor status or *HER2* amplification suggesting that it may be a novel target for triple negative breast tumors (Table 1). A strong association was noted between SUSD2 and CD68 staining, indicating that SUSD2 attracts macrophages into the TME (Fig 1). Furthermore, the M2 phenotype comprised the majority of TAMs present in tumors that had strong staining for SUSD2 (Fig 2). M2 polarized TAMs are known to promote angiogenesis, providing further support that increased TAM recruitment and angiogenesis is a consequence of *SUSD2* expression in these tumors. In order to support this data, an *in vitro* co-culture assay was developed. Macrophages that were co-cultured with MDA-MB-231-SUSD2 showed twice as many M2 macrophages compared to those co-cultured with MDA-MB-231-vector. This experiment provides additional evidence that SUSD2 increases the M2 polarized macrophages in the tumor microenvironment. Future studies will focus on determining the mechanism of this M2 polarization. We hypothesize that these results may be explained by the interaction of SUSD2 with Galectin-1, a protein known to diminish M1 macrophage activation and support M2 activation [35,36]. Our lab has previously demonstrated that Galectin-1 is only presented on the surface of BCa cells when SUSD2 is present (24); therefore, SUSD2 presentation of Galectin-1 on the cancer cell membrane may allow direct regulation of macrophage phenotype upon cell contact.

Low-Marchelli *et al*. proposed a model of angiogenesis involving TAM recruitment mediated by MCP-1 [22]. Consistent with that model, our data indicated that high levels of SUSD2 in BCa cells have a direct correlation with the increased amount of MCP-1. As shown by RT-qPCR, *SUSD2-*expressing BCa cells had up-regulated levels of *MCP-1* compared to vector control cell lines (Fig 5A). Moreover, intracellular MCP-1 protein levels directly correlated with increased MCP-1 secretion in cell culture supernatants (Fig 5B, 5C and 5D). Additionally, increased levels of MCP-1 were observed in *Susd2*-expressing mammary tumors isolated from our isogeneic mouse model (Fig 5E). Since SUSD2 increases secretion of MCP-1 and secreted MCP-1 recruits macrophages, one would predict that patient tumors with strong SUSD2 staining should contain more macrophages than tumors that have weak to no SUSD2 staining. Data shown here using both patient and mouse tumor samples strongly supports this hypothesis. Our data indicates that patient tumors with strong SUSD2 staining had increased CD68 staining of macrophages in the tumor (Fig 1A and 1B). This correlation of increased TAMs with high levels of Susd2 was also shown in mouse tumors using the *in vivo* model (Fig 1C).

SUSD2 also promotes angiogenesis independent of TAM recruitment. CM harvested from *SUSD2*-expressing cells increased tubule formation of HUVEC cells nearly 2-fold compared to CM harvested from cell lines with low levels of SUSD2 for both MDA-MB-231 and SKBR3 cell lines (Fig 6). No cell-to-cell contact was needed for HUVEC stimulation indicating that *SUSD2*-expressing cells secrete angiogenic factors that directly stimulate endothelial cells to form vessels in tumors.

Angiogenesis is a complex physiological process that is hijacked by cancer cells to create an environment conducive to tumor growth. This process involves the manipulation and reprogramming of non-malignant cells, such as TAMs, to promote neovascularization in tumors. Here we show that *SUSD2*-expressing BCa cells potentiate angiogenesis indirectly by the recruitment of macrophages into the tumor by secretion of MCP-1 and by secreting angiogenic factors that directly stimulate endothelial vessel formation. Further, SUSD2 promotes the polarization of TAMs to the M2 phenotype. Since SUSD2 is a membrane protein that is abundant in 80% of patient BCa samples, further study of this pathway may lead to the identification of novel therapeutic targets to inhibit the function of SUSD2 and ultimately impede cancer metastasis.

## Supporting information

S1 Fig Immunohistochemical analysis of SUSD2 in breast cancer cell lines. To verify *SUSD2* expression and localization in the generated cell lines used in our study, MDA-MB-231-SUSD2 and -vector control cell lines as well as SKBR3-NT and *SUSD2* knockdown cell lines were grown in culture, pelleted, fixed and paraffin embedded. Sections were analyzed with immunohistochemistry using an anti-SUSD2 antibody. Positive staining is indicated by the brown color. Cells were counterstained with hematoxylin. MDA-MB-231-vector and SKBR3 KD1-4 showed very weak SUSD2 staining. SKBR3 KD4-4 had moderate SUSD2 staining indicating that SKBR3 KD1-4 is a more complete knockdown. MDA-MB-231-SUSD2 and SKBR3-NT cell lines showed robust staining of SUSD2 on the cell membrane. Images were taken at 200x.
(TIFF)
