# Supplementary figures and images for: SUSD2 promotes tumor-associated macrophage recruitment by increasing levels of MCP-1 in breast cancer
Source: PLoS One. 2017 May 5;12(5):e0177089. doi: 10.1371/journal.pone.0177089 (PMC5419604; doi:10.1371/journal.pone.0177089)

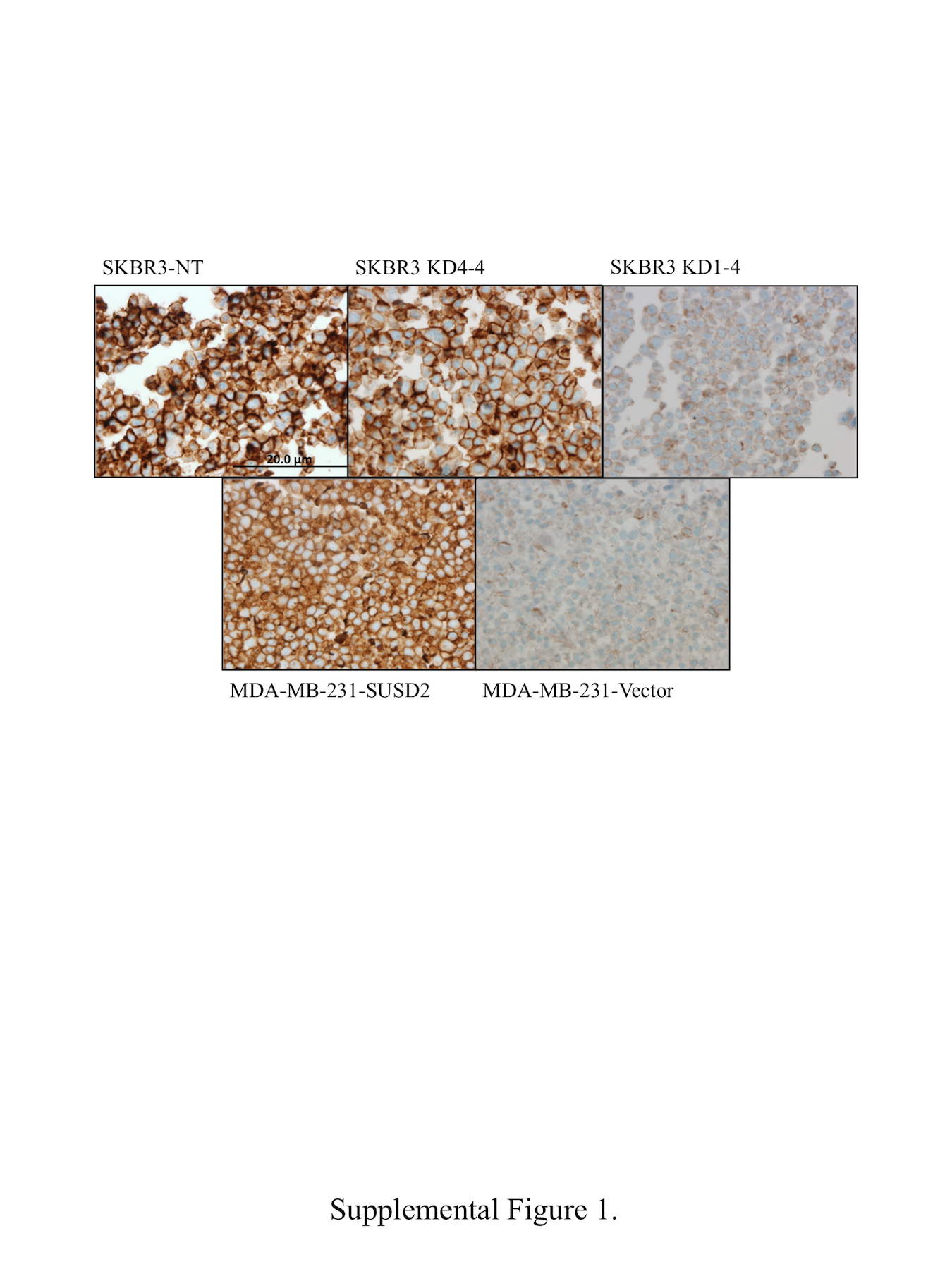

Supplement: S1 Fig — To verify SUSD2 expression and localization in the generated cell lines used in our study, MDA-MB-231-SUSD2 and -vector control cell lines as well as SKBR3-NT and SUSD2 knockdown cell lines were grown in culture, pelleted, fixed and paraffin embedded. Sections were analyzed with immunohistochemistry using an anti-SUSD2 antibody. Positive staining is indicated by the brown color. Cells were counterstained with hematoxylin. MDA-MB-231-vector and SKBR3 KD1-4 showed very weak SUSD2 staining. SKBR3 KD4-4 had moderate SUSD2 staining indicating that SKBR3 KD1-4 is a more complete knockdown. MDA-MB-231-SUSD2 and SKBR3-NT cell lines showed robust staining of SUSD2 on the cell membrane. Images were taken at 200x. (TIFF) [file pone.0177089.s001.tiff]
